# Supplementary material for: Dynamics of major plant nutrients and enzymatic activities in soil influenced by application of biochar and organic waste
Source: PLoS One. 2024 Oct 30;19(10):e0307487. doi: 10.1371/journal.pone.0307487 (PMC11524486; doi:10.1371/journal.pone.0307487)
Supplement: Attachment — Submitted filename: Review report PLOS.doc [file pone.0307487.s001.doc]

**I would like to express my gratitude for providing me with the opportunity to review the article. However, while reviewing this article I have found few suggestions which are given below:**

1. As you mentioned, nine modules of fertility have: been examined in the study. Please clarify the which kind of module you have tested under the study. The word use module is appropriate in this present study; clarify?
2. It was a single year field experiment, and could you maintain the consistency of the results on a long-term basis?
3. Need to include the kinetics of different organic materials (biochar, FYM, and pressmud) used in experimentation in saline soil.
4. The three organic materials used in the experiment were applied at the same rate. It is important to clarify whether their nutritional compositions are the same or different. The basis for using the equal rate of application needs to be addressed with justification.
5. The abstract stated that the addition of organic substance (10 t/ha) did not affect the soil pH. However, the results show that the application of different organic and inorganic treatments significantly affected the soil pH. These statements are contradictory. Please clarify.
6. Applied treatments have been used to enrich the different nutrients, then how could you compare the treatments for the studied growth parameters without bias?
7. The "Discussion" section needs to be strengthened with appropriate findings and justifications.
8. The conclusion of the manuscript needs to be revised and concise, focusing on the findings of the results.

To define the sustainability of any practices, they have to be evaluated for long-term study at multiple locations. Addressing these concerns is crucial before considering the article for publication.
