# Supplementary material for: Dynamics of major plant nutrients and enzymatic activities in soil influenced by application of biochar and organic waste
Source: PLoS One. 2024 Oct 30;19(10):e0307487. doi: 10.1371/journal.pone.0307487 (PMC11524486; doi:10.1371/journal.pone.0307487)
Supplement: Attachment — Submitted filename: Response letter to Editor.doc [file pone.0307487.s002.doc]

Response to PONE-D-24-12566 (Mineralization kinetics of major plant nutrients in saline soil influenced by application of biochar and organic waste).

| **Correction** | **Reply** |
| --- | --- |
| Academic Editor comments | |
| Thank you for submitting your manuscript to PLOS ONE. After careful consideration, we feel that it has merit but does not fully meet PLOS ONE’s publication criteria as it currently  stands. Therefore, we invite you to submit a revised version of the manuscript that addresses the points raised during the review process. | Thank you for kind words. Manuscript was modified as per the direction of Reviewers. |
| **Reviewer 1** |  |
| The research question is not completely clear throughout the introduction, which also lacks a clear hypothesis. Please present a hypothesis. Remember that the research objectives must result from the research hypothesis. | Agreed; Modified the MS as per your directions  Modified |
| Please, could you clarify the use of @ symbol to express the different treatments applied? I suggest removing it and replacing it | Corrected |
| A deeper scientific interpretation of your findings in the results and discussion section is strongly suggested. The novelty and implication of your study should also be highlighted throughout this section. Paragraphs should be better structured and interconnected. The discussion should link all of your findings. | Thoroughly revised |
| A systematic description of your results must be done, highlighting for the reader observations that are most relevant to the topic under investigation and it can be used in the discussion later. | Thoroughly revised |
| The conclusions section should illustrate the mechanistic links of findings obtained under applied treatments. The authors should avoid repeating what has already presented in results and discussion. Please, avoid using abbreviations and acronyms in the conclusions section. Remember that the conclusions must be self-explanatory. | Thoroughly revised |
| **Specific comments** |  |
| What is the method of application of biochar and organic waste? | Added in M & M |
| What is the moisture content of soils thought out the incubation study? | Added in M & M |
| How to prepared pigeon pea biochar. Mention method and conditions. |  |
| In general, biochar is alkaline in nature but in this study the pH of biochar is 7.6. Why? | Extremely sorry sir, it was typing error . we have checked the analysis. Data, corrected in MS. It is alkaline in nature |
| What is QA/QC…expand it | Incorporated |
| Mention TOC and nutrient composition of pressmud. | Incorporated |
| Figure 1 needs some correction. | Modified |
|  |  |
| In page 1, SOM and C… Elaborate first time appeared | Added |
| In the abstract and table 2, it is mentioned that the addition of organic substances (10 t/ha) didn’t affect the soil pH, whereas in the result and discussion section, it is vice versa  (the application of different organic and inorganic treatments significantly affected the soil pH). | Checked and revised |
| The overall quality of the MS could be enhanced after addressing those issues | Thoroughly revised as directed by reviewers suggestions |
| **Reviwer 2** | |
| MS" Mineralization kinetics of major plant nutrients in saline soil influenced by application of biochar and organic waste" falls under the aim and scope of the journal. it  is written in scientific way.  it is having minor suggestions | Thank you for valuable comments |
| Revised tile as per the study finding. | Modified |
| strengthen the M&M part | Added |
| Please check reference list | Checked, it is okay |
|  |  |
| Reviewer #3 |  |
| Identify research gaps and highlight importance of the present investigation.  Specify clearly objective of the investigation. | Revised |
| Add this para in introduction  Combined use of NPK along with FYM and Pressmud improved soil nutrient status, organic carbon fractions and carbon management index under pigeonpea-wheat cropping system (Mandal et al., 2013) and soil hydro-physical properties (Mandal et al., 2018). FYM, VC along with PSB also solubilized P from Fe-P and Al-P (Bairwa et al., 2021). | Added in Introduction and in Discussion part |
| Materials and Methods  Section 2.3 : Mention details of incubation temperature and moisture content of soil maintained during laboratory incubation experiment. | Added |
| Conclusions  Revise conclusion part. Highlight specific recommendation in quantitative terms. Give future line of work in this direction | Revised |
|  |  |
| Reviewer #4: |  |
| Revise the manuscript, considering the given suggestions. If possible, then include more data and a sound discussion to justify the requirement of a good quality publication. Critically check the abstract and conclusion; it has some contradictory statements. | Revised |
|  |  |
| Reviewer #5: |  |
| The authors have used Mineralization kinetics word in the manuscript title, however there is no kinetics involved in result or conclusion. The title misleads that first or second order kinetics has been for mineralization study. Further, the results has no data on mineralization or nutrient dynamics. The results of incubation study presented is only for the change in initial and final concentration of NP S and OC. Its a simple experiment with no new information as its a well known fact that addition of organic matter in any form would certainly enhance nutrient content in soil especially when there is no crop under study ie an incubation experiment. | Extremely sorry for inconvenience  MS thoroughly revised |

Changes in MS is in track change mode

Kindly acknowledge the receipt of the same.

Warm Regards

Dr M L Dotaniya
